# The Potential to Forgo Social Welfare Gains through Overrelianceon Cost Effectiveness/Cost Utility Analyses in the Evidence Base for Public Health

**DOI:** 10.1155/2009/107927

**Published:** 2009-12-02

**Authors:** D. R. Cohen, N. Patel

**Affiliations:** Health Economics and Policy Research Unit, University of Glamorgan, Pontypridd, CF37 1DL, UK

## Abstract

Economic evaluations of clinical treatments most commonly take the form of cost effectiveness or cost utility analyses. This is appropriate since the main—sometimes the only—benefit of such interventions is increased health. The majority of economic evaluations in public health, however, have also been assessed using these techniques when arguably cost benefit analyses would in many cases have been more appropriate, given its ability to take account of nonhealth benefits as well. An examination of the nonhealth benefits from a sample of studies featured in a recent review of economic evaluations in public health illustrates how overfocusing on cost effectiveness/cost utility analyses may lead to forgoing potential social welfare gains from programmes in public health. Prior to evaluation, programmes should be considered in terms of the potential importance of nonhealth benefits and where these are considerable would be better evaluated by more inclusive economic evaluation techniques.

## 1. Introduction

Evidence-based medicine is now an established paradigm within health care [1]. Growing recognition that resources for health care are scarce has led to broad acceptance that the evidence base should include economic as well as clinical evidence. In the UK, this is reflected in the work of the National Institute for Health and Clinical Excellence (NICE) whose national guidance on health care for England and Wales is explicitly informed by evidence of cost effectiveness as well as clinical effectiveness [2]. 

The principal benefits from most clinical treatments are in the form of health gains to patients. While there can also be nonhealth benefits, for example, from earlier return to work or in terms of reduced burden on informal carers, these tend to be relatively small. Since an objective of all health care systems is to maximise the amount of health produced (allowing for other objectives such as equity), it is not surprising that the economic questions most commonly addressed when evaluating clinical treatments are concerned with identifying the most cost-effective ways of producing health. 

There is now a growing movement toward incorporating the principles of evidence-based medicine in evidence-based public health. At the end of 2005, NICE announced that it was extending its remit to include guidance on the promotion of good health and the prevention of ill health [3] thus explicitly recognising the contribution of public health in improving health. 

A review of economic evaluations in public health was recently undertaken [4]. A total of 1697 papers met the review's inclusion criteria. Of these, 1235 (73%) were classed as cost effectiveness (CEA) or cost utility (CUA) studies which assess how 68 cost effectively a public health programme produces health. Only 43 studies (2.5%) were classed as cost benefit analyses (CBA)—the technique of economic evaluation which addresses the broader issue of whether or not a public health programme is *worthwhile*. 

This paper considers the fundamental differences between CEA/CUA and CBA in the context of providing an evidence base to inform public health policy. Using selected studies from the database produced by the team that undertook the review [4], this paper examines how information provided by CEA/CUA studies might lead to inefficient recommendations for public health policy. It does this by briefly reviewing the principles of the different techniques of economic evaluation, considers the fundamental differences between public health and clinical treatments in terms of their objectives, and then, on the basis of information from a selection of studies, suggests a simple method for assessing where CEA/CUA of public health programmes might make a misleading contribution to the economic evidence base for public health.

### 1.1. Principles of Economic Evaluation of Clinical Treatments

Within health care, a cost effectiveness analysis will assess an intervention against a comparator in terms of cost per unit of health effect achieved. These units can be specific, for example, true positive cases of presymptomatic disease detected in a screening programme, or more generic, for example, life years saved. In cost utility analysis, the unit of effectiveness is the Quality Adjusted Life Year (QALY) or other single index measures which capture both life length of life and quality of life. The technique's name refers to the fact that the quality of life element is determined by the utilities, or values, attached to different health states. Thus while CUA outcomes take account of preferences they can still be regarded as a form of CEA since they seek to find the least cost way of producing a health-related unit of effect. NICE determined early on that, where possible, CUA was the preferred form of analysis to provide the economic evidence for health care interventions [2]. 

CEA/CUA, however, only compare alternatives ways of pursuing a single objective—in this case to maximise health gain. An unambiguous result, however, is produced only if the intervention in question is both more effective and less costly than the comparator (or vice versa). It is then said to be “dominant” and there are no economic arguments for not adopting it over the comparator. 

A far more common result, however, is one of nondominance. This occurs where the intervention of interest is more effective but also more costly. Whether or not the higher costs are *worth* incurring, that is, whether additional resources should be *allocated* to the treatment of these patients is an *allocative *efficiency question which cannot be answered by CEA/CUA. 

In order to assist decision makers, nondominant results are commonly presented in the form of incremental cost effectiveness ratios (ICERs) which show the extra cost of achieving the extra health effects. Cost effectiveness acceptability curves (CEACs) deal with the uncertainty surrounding the estimates by showing the probability that a nondominant intervention will have an ICER below a range of thresholds which represent the maximum amounts that a payer would be willing to pay for an extra unit of effect [5]. 

Heuristics are often used to identify these thresholds. For example, NICE currently regards an ICER of *£*30 000 per QALY as being at the upper limit for the interventions to be recommended for use in the British National Health Service. While the question of how much society is willing to pay for extra health benefits remains a live issue, the focus on assessing the cost effectiveness of clinical treatments through CEA/CUA remains the norm. 

Results of these analyses clearly depend on which costs, including cost savings, are included, that is, on the perspective adopted. In the UK, NICE restricts the perspective to the National Health Service and personal social services. Costs (positive and negative) to other sectors are not considered—at least in the primary analysis. This remains a contentious issue as exemplified in a recent NICE appraisal of drugs for Alzheimer's disease where a major issue was whether or not to include cost borne by informal carers [6] and a recent editorial in the British Medical Journal has called for a rethink on continued use of a narrow perspective even for health service interventions [7].

### 1.2. Principles of Economic Evaluation in Public Health

The UK Faculty of Public Health has adopted Sir Donald Acheson's definition of public health as “the science and art of preventing disease, prolonging life and promoting health through organised efforts of society” [8]. The Faculty regards the key elements of public health as being population based, emphasising collective responsibility for health, recognising the key role of the state, and emphasising partnerships with all those who contribute to the health of the population. These principles make it clear that public health programmes can produce health in ways which do not necessarily involve health professionals or involve the use of health services. 

While these principles are captured in most definitions of public health, the definition by Allin et al. ends with “*…*and involves mobilising local, regional, national and international resources to *create conditions in which people can be healthy”* (emphasis ours) [9]. In contrast to producing health by treating illness, creating the conditions in which people can be healthy will often achieve important nonhealth benefits in addition to health. Evidence that such nonhealth benefits are positively valued was demonstrated in a study by Cropper [10] which examined people's preferences for different life saving programmes. When asked to choose between programmes, a belief that a programme would produce benefits in addition to life saving was shown to significantly increase the probability of that programme being preferred over another with the same life saving benefits. 

Cost benefit analysis (CBA) is the technique of economic evaluation which addresses *allocative* efficiency. It explicitly addresses the question “how much more or how much less of society's resources should be allocated to achieving this goal or to this type of healthcare?” [11]. Its foundations are rooted within welfare economics where the aim is to assess how social welfare is affected by a particular project [12]. CBA does this by identifying and measuring all costs and all benefits; defined as everything of value that results (positive or negative) and regardless of who gains or loses, that is, using a *societal perspective*. When all gains and losses are measured in commensurate terms (i.e., money) healthcare objectives can be compared with each other or with those in other sectors of the economy. If the total value of the benefits (gains) exceeds the total value of the costs (losses), then the proposal passes the cost benefit test and total social welfare is increased by implementing the programme. 

Although in principle CEA/CUA are capable of capturing avoided costs in sectors other than health care, these analyses would still not capture the full range of benefits that would be picked up in a CBA. For example, a CUA of a proposed policy to reduce air pollution would be based on the narrow premise that the objective of the policy is solely to produce health. Thus, the benefit side would include only health gains. If the analysis showed an incremental cost/QALY above a predetermined threshold, even if that analysis included cost savings to sectors other than health such as reduced cleaning costs due to reduced pollution, the implication would be that resources should not be allocated to this intervention. A CBA, however, which included nonhealth as well as health benefits—say the value attached to breathing clean air independent of any health implications—might show the same proposal to pass the cost benefit test. In this example, a policy decision taken on the basis of the CUA would mean forgoing an opportunity to improve social welfare. It is, of course, possible that nonhealth benefits could be negative which reinforces the importance of not excluding them, particularly where they are significant. 

Although economists have developed numerous methods to assign money values to costs and benefits which do not have associated market prices, this inevitably is not an easy task which might explain why comprehensive CBAs remain rare. It is well recognised that many studies which include the word “cost benefit analysis” in their title are, in reality, not CBA studies at all [13]. Such mistitled studies frequently use that term because they regard cost savings from avoided future illness as ‘benefits’ and thus feel that their analysis has covered both sides of the cost benefit calculus. 

Some economic studies avoid valuation problems by simply listing the costs and consequences of any activity without aggregation. Such cost-consequences analyses (CCAs) are, strictly, not economic evaluations as they cannot provide answers to either cost effectiveness or allocative efficiency questions. However, by identifying costs and consequence they can be an important aid to decision making beyond cost effectiveness ratios and a recent report from the Public Health Research Consortium in the UK has called for the intersectoral impacts of public health interventions to be presented in the form of a cost-consequences analysis [14]. 

In terms of an emerging public health evidence base, there thus appears to be a problem. If the benefits from public health interventions frequently include more than just health gains then, arguably, its evidence base ought to include a smaller proportion of cost effectiveness and cost utility analyses than does the evidence base for clinical treatments. Nevertheless, as shown in the review [4], nearly three quarters of the economic evaluation undertaken in the area of public health have to date been cost effectiveness or cost utility studies. 

In order to illustrate the partial nature of the information provided from CEA/CUA evaluations in public health, hence the potential for inefficient health policy, the present study examined the nature and relative importance of benefits which were included in some of the economic studies which went beyond cost effectiveness or cost utility analyses in the recent review [4].

## 2. Materials and Methods

Studies from the review were identified for possible selection if they were not classed as cost effectiveness or cost utility studies. It was evident from examination of abstracts, however, that despite their classification many of these studies had limited their analyses to health benefits alone. Omission of nonhealth benefits, even where they might be significant, is not necessarily a weakness in these studies. If the value of the health benefits alone can be shown to exceed the value of all of the costs, then the intervention passes the cost benefit test without the need to consider any nonhealth benefits. Including them would only reinforce the conclusion already reached although clearly, incomplete assessment would make comparisons of the benefit : cost ratios between programmes problematic. Given the focus of the present exercise, these studies were ignored. 

Ten studies were selected for illustration (Table 1). Of these two had been classed within the review as CBA; the remainder being CCA or multimethod apart from one case where no classification was given. 

## 3. Results

The work by Aunan et al. [15] evaluated the costs and benefits of implementing to reduce air pollution programme. Reduced damage to public health, building materials, and agricultural crops from reduced emissions of air pollutants was assessed over two medium term targets (5 years and 10 years). The possible benefits from implementing the measures described by the National Energy Efficiency Improvement and Energy Conservation Program were evaluated using saved energy from various sectors: households, transportation, industry, service, energy, and agriculture. 

Health benefits included those from acute respiratory symptoms, chronic respiratory symptoms, infant deaths, and lung cancer. Nonhealth benefits included damage to materials through atmospheric corrosion and deterioration of materials. This included the maintenance and replacement costs from reducing SO_2_ concentration levels. Crop loss due to SO_2_ was seen to be a great concern for crop production. 

The analysis indicated that the annual benefit of improved health alone is likely to exceed the investment needed to implement the programme. Thus, the policy would pass the cost benefit even without inclusion of the significant benefits due to reduced damage to materials and crops. 

The work by Miller et al. [16] modelled the potential health and economic impacts of implementing a medically prescribed heroin programme among Canadian injecting drug users over 5 years. The potential impact of the programme was estimated by comparing hospitalisation and emergency use costs. Nonhealth costs in the study included criminal activity and productivity losses. 

Benefits were measured by the reduction in these costs. Reductions in criminal activity costs accounted for fully 63% of the total reduction in costs. Other costs avoided since the implementation of the programme such as the costs of social housing, use of social services, counseling, and employment programs were identified but not included in the model.

Although this study did not claim to be a CBA it provides an example of an intervention whose nonhealth benefits were significantly larger than the health benefits. Had the researchers attempted a CBA, the nonhealth benefits could have made the difference between the programme passing or failing the cost benefit test.

The work by Zeng-Sui et al. [23] assessed the impact on enteric infectious disease by providing deep-well tap water across six villages in China. Health benefits included reductions in diarrhoea, dysentery, viral hepatitis, cholera, and reduce mortality form liver cell cancer. Nonhealth benefits included reductions in lost wages or earnings of patients and of their relatives who looked after them during the illness and the avoided costs of transportation, supplemental nutrition, and the value of gifts sent by relatives to assist towards their recuperation (but interestingly not the value of the gift to the recipient which illustrates how the gifts should have been regarded as a financial transfer [25] rather than an economic cost avoided). 

The study did not include all health benefits. For example, water-related conditions such as skin and eye infections, dermatosis, gynaecological conditions, parasitic enteric diseases, and vector-borne diseases were mentioned but not included. Other intangible benefits such as the improved service that will benefit future generations were also mentioned but not included in the analysis. Overall assessed benefits were more than double the costs.

The work by Guria et al. [20] evaluated the incremental outcomes of road safety programmes and driving campaigns enforced in New Zealand and compared them with their resource costs. In addition to loss of life and reduced quality of life resulting from injury, the study also included the social costs of injuries and property damage avoided. Other benefits such as the development of a safety culture, improvement of road user behaviour, and the safety quality of vehicles were mentioned but not included. 

The study showed that road safety programmes aimed at reducing high-risk behaviours produced high returns. If 90% of road safety expenditure is attributed to the period of investment, then the benefit to cost ratio would be 12.3 : 1 for 1993–1995 and 7.9 : 1 for 1994–1996.

The work by Aehyung et al. [21] presented a CBA of the onchocerciasis (river blindness) control programme. Health benefits included the number of cases of blindness and death prevented. Nonhealth benefits included additional agricultural output as a result of a more productive labour force and additional agricultural land made available through the control of onchocerciasis. Other nonhealth benefits such as the reduction of lost production time by family members when providing care and improved parenting were mentioned but not included in the study. 

The study showed that the land related benefits were large. The net present value (NPV) ranged from US$485 million to US$3,792 million (1987 dollars) depending on the assumptions used. A positive NPV is another way of saying that the programme passed the cost benefit test.

The work by Fleming et al. [19] estimated the costs and benefits of brief physician advice with problem drinkers in primary care settings. Health care benefits included avoided cost from the perspective of the managed care organisation, the use of equipment, personnel, emergency medical care, hospitalisations, treatments, and clinic visits. Nonhealth benefits included legal events and motor vehicle accidents. 

The study indicated that physician-delivered advice can reduce not only medical costs but also social costs associated with alcohol consumption. The total economic cost of the intervention was $80,210, or $205 (1993 dollars) per study patient. The total benefit of this brief physician intervention was $423,519. The total benefit was equal to $1,151 per study patient.

The study by Cohen et al. [18] estimated the potential benefits from saving a “high-risk youth” by estimating the lifetime costs associated with the career criminal drug abuser and high school drop out. Antisocial behaviour of career criminals was included as an externality and thus seen as imposing as a social cost. Assessed nonhealth benefits included the avoided social costs from stolen property and lost wages. 

As this study did not examine the costs of interventions aimed at reducing antisocial behaviour, it was not a CBA. However, the range and magnitude of the nonhealth benefits which were included in the valuation exercise illustrates what would be missed if a CEA/CUA study focussing solely on the health benefits had been undertaken.

The work by Caulkins et al. [17] on school-based drug prevention programmes focused on reducing drug consumption, particularly cocaine, as an objective of the nation's drug control efforts. The study reported the quantity of cocaine consumed, the cost of drug use, and the social value of cocaine control. 

Benefits from the prevention program included reductions in the use of other drugs including marijuana, alcohol, and cigarettes. Nonhealth benefits included lower crime rates, higher productivity, and an increase in the number of pupils graduating from high school graduation. The programme was deemed to be affordable and social benefits were shown to exceed the total costs which justified implementation of the programme.

The work Ginsberg et al. [24] estimated the costs of making the wearing of bicycle helmets compulsory in Israel. Benefits included resource saving from fewer head injuries in terms of hospitalisation, emergency room visits, ambulatory care, rehabilitation, long-term care, and special education. Also included were productivity benefits and the value of avoided death. 

The authors called their analysis “conservative” as “*…*it did not consider reduced pain, worry, grief, work losses for ambulatory visits or even time off from housework as a result of bicycle injuries. Nor did we consider the intangible benefits of the lessening of anxiety concerning crashes by cyclists or by their friends and relatives.” 

Inclusion of these additional benefits was unnecessary as the partial benefits (total = US$60.7 million) clearly exceeded total costs (US$20.1 million) without their inclusion (dollar base year not stated). In this example, the health service savings alone (US$44.2 million) were sufficient for the proposed policy to pass the cost benefit test. If, however, the results showed that the productivity gains (US$7.5 million) were needed to tip the balance, then their omission would have meant a lost opportunity to increase social welfare.

The work by Miller et al. [22] examined counselling by paediatricians aimed at reducing injuries in children. Benefits included savings from hospital admissions, professional services, rehabilitation, prescriptions, home health care, and medical equipment. The study also included benefits from avoided productivity losses due to children not being able to work when they became adults if they were killed or permanently disabled and included a value for avoided pain, grief, and suffering. 

The study was acknowledged to be only partial. For example, it mentioned but did not include productivity losses from parents dealing with injured children. Nevertheless, it still showed a benefit to cost ratio of nearly 13 : 1. In this case, however, annual productivity benefits ($660 million) were nearly 3 times those of health service benefits ($230 million) (1992 dollars) which in a higher-cost programme might have made the difference between passing or failing the cost benefit test.

## 4. Discussion

It is evident that many public health interventions produce benefits in addition to health and in many cases these can be substantial. Some of these involve resource savings which, if regarded as negative costs could in principle be included in a CEA/CUE provided that a societal perspective were adopted. Others, however, for example, reductions in criminal activity, are clearly of value independent of any cost savings and these would not feature in a cost effectiveness or cost utility study. 

The extent to which the noninclusion of nonhealth benefits in evaluation by CEA/CUA represents a problem—in the sense that it could potentially lead to foregone opportunities to increase social welfare—might be predicted by considering where the intervention in question would sit along a continuum of intent. 

At one extreme of such a continuum would be public health measures whose intent was solely to produce health. For example, a policy to add folic acid to flour has been advocated with the specific intent of reducing the incidence of neural tube defects (NTD) in newborns [26]. Where health gains are the sole objective of a public health programme then, on the same principles used within health care, they can be assessed in terms of cost effectiveness. If the addition of folic acid to flour were shown to have a low incremental cost/QALY, then this public health measure would be a cost-effective way of producing health relative to interventions within health care. 

Even in this example, however, there could still be a case for directly addressing allocative efficiency through CBA, for example, if women of childbearing age receive immediate reassurance from the knowledge that eating fortified food reduces their risk of conceiving a baby with an NTD. Equally, making consumption of folic acid compulsory removes freedom of choice which to many could be a highly negatively valued nonhealth outcome. 

Moreover, although there are no rules within the methodology of economic evaluation to prevent a CUA being undertaken from a broad perspective––and there have been recent calls to do just that [7]—such evaluations remain uncommon. In the folic acid example, the cost/QALY derived from a study which adopted a health service perspective would not include savings to other agencies such as special education which in the case of children with NTD could be substantial. 

Further along the continuum of intent would be public health interventions where health is the primary concern but other objectives will clearly also be achieved. Thus, while a road safety intervention may be advocated primarily to reduce injury and death on the roads, it is evident that a reduction in accidents will also produce savings in terms of property damage. The study by Guria et al. [20] on road safety in New Zealand included property damage in deriving its cost benefit ratios. 

Further still along the continuum would be public health interventions which clearly address multiple objectives. For example, illegal drug use is known to cause many social problems as well as health problems. Thus the study by Caulkins et al. [17] included reduced crime and increased productivity among the benefits of schools-based drug prevention programmes, even taking account of intangible benefits such as an increase in the proportion of pupils graduating from high school. Another example is the onchocerciasis prevention programme examined by Aehyung et al. [21] which, although driven by a desire to reduce the incidence of river blindness, would also free previously oncho-ridden tracts of land for settlement and cultivation. In both these examples, CUAs would have given misleading information for policy. 

Further still along the continuum would be programmes which could be perceived as being only incidentally preventive in the sense that the effect of pursuing another policy objective would incidentally have a positive effect on health. An example here could be improvements in housing which are undertaken to provide people with more pleasant places to live but which can at the same time affect respiratory illnesses or reduce injuries. The health effects of such programme can be assessed via a “health impact assessment.” 

Ultimately almost any public policy can be seen as containing an element of public health. For example, macroeconomic policies to stimulate economic growth are clearly driven by concerns other than health, yet economic growth reduces unemployment and the relationship between unemployment and ill health is long established [27].

## 5. Conclusion

Public health programmes can have nonhealth benefits which may not be captured when a cost effectiveness/cost utility approach to economic evaluation is undertaken. Omission of nonhealth benefits could mean forgoing opportunities to improve social welfare. A preanalysis examination of where any public health intervention would be located on a continuum of intent (relative importance of health versus nonhealth benefits) could identify where evaluation by cost effectiveness or cost utility analysis might produce inappropriate conclusions for policy. 

Many of the public health programmes which to date have been assessed by CEA/CUA, in particular, those addressing smoking cessation [28] have shown incremental cost effectiveness ratios which are far below current thresholds. In such cases, the use of more comprehensive techniques is unnecessary. Equally, where a CBA is undertaken and the value of the health benefits alone is anticipated to clearly outweigh the costs, addition of nonhealth benefits will not affect the decision on whether to implement the programme and hence their inclusion is again unnecessary. Most public health programmes, however, are unlikely to allow such obvious a priori conclusions to be drawn and it is here that consideration of where the programme sits along the suggested continuum of intent will increase the likelihood that the most appropriate technique of economic evaluation will be used. 

## Figures and Tables

**Table 1:** Selected studies from review [4] database.

Author	Year	Title	Country of study	Area of Study	Evaluation method	Nonhealth benefits measured
Aunan et al. [15]	1998	Health and environmental benefitsfrom air pollution reductions in Hungary	Hungary	Pollution / Toxicity	CCA	Avoided damage to materials, crops and vegetation; climate change

Caulkins et al. [17]	1999	An ounce of prevention, a pound of uncertainty:The cost-effectiveness school-based drug prevention programs	USA	Drugs and alcohol	Multitype	Reduced crime rates; higher productivity; increase in numbers graduating high school

Cohen MA et al. [18]	1998	The monetary value of saving a high-risk youth	USA	Health Promotion	Not classified	Reduced victim costs of crime; savings to criminal justice system

Fleming et al. [19]	2000	Benefit-cost analysis of briefphysician advice with problem drinkers in primary settings	USA	Drugs and alcohol	CCA	Savings in lost wages, transportation, legal events, motor vehicle accidents and crime

Guria et al. [20]	1998	An economic evaluation of incremental resources to road safety programmes in New Zealand	New Zealand	Drugs/ Alcohol	CBA	Reduced property damage

Aehyung et al. [21]	1995	Cost Benefit Analysis of theOnchocerciasis Program	Africa	Disease and Infection	CCA	Additional agricultural output and freed land for productivity

Miller C.L et al. [16]	2004	The potential health and economicimpact of implementing a medicallyprescribed heroin program among Canadian injection drug users	Canada	Drugs and Alcohol	Multi type	Increased employment, reduction in criminal activity

Miller T.R et al. [22]	1995	Injury Prevention Counselling by Paediatricians: A Benefit- Cost Comparison.	USA	Health Promotion	CBA	Savings from professional services, rehabilitation, avoided productivity losses

Zeng-Sui et al. [23]	1989	Reduction of enteric infectiousdisease in rural China by providing deep-well water	China	Disease and Infection	CCA	Lost wages,

Ginsberg et al. [24]	1994	A cost- benefit analysis of legislation for bicycle safety helmets in Israel	Israel	Injury Prevention	CBA	Increased productivity, savings in special education
